# Supplementary material for: Pathophysiological Significance of Neutrophilic Transfer RNA-Derived Small RNAs in Asymptomatic Moyamoya Disease
Source: Cells. 2021 May 1;10(5):1086. doi: 10.3390/cells10051086 (PMC8147334; doi:10.3390/cells10051086)

| 2D Structure | Local AU | Position |
|---|---|---|

```
781                           811
                        8mer-1a
 5'-ttcttttTAATTAGATAAATCAC-ACGGAAAa-3' utr
            |||::|  ||   ||| |||||||
 3'-uuugcacuAUUGGU-GAU--GUGAUGCCUUUUg-5' tRF
              16 151413     7 6 5 4 3 2
           3' Pairing          Seed
```

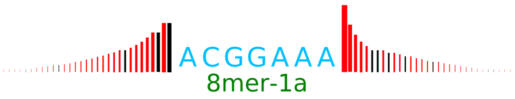


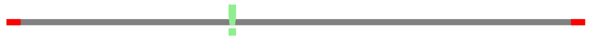

Supplement: Supplementary file 1 [file cells-10-01086-s001.zip › cells-1149161 3rd supplementary materials/Data Supplements/Data Supplement V.zip!/Data supplement 6-mRNAs targets of tsRNAs/tsRNA-Val-AAC-017/Diagrams/tRF-Val-AAC-017_vs_ENST00000322652(SUZ12).pdf]
